# Effectiveness and Safety of Balloon Pulmonary Angioplasty for the Treatment of Patients with Persistent Pulmonary Hypertension after Pulmonary Endarterectomy

**DOI:** 10.3390/jcm12030905

**Published:** 2023-01-23

**Authors:** Nicolas M. Maneiro Melon, Maite Velazquez Martin, Sergio Huertas Nieto, Agustin Albarran Gonzalez-Trevilla, Fernando Sarnago Cebada, Alejandro Cruz Utrilla, Williams Hinojosa Camargo, Ricardo Aguilar Colindres, Maria Melendo Viu, Maria Jesus Lopez Gude, Rafael Morales Ruiz, Marta Perez Nuñez, Fernando Arribas Ynsaurriaga, Pilar Escribano Subias

**Affiliations:** 1 Cardiology Department, Hospital Universitario 12 de Octubre, 28041 Madrid, Spain; 2 Instituto de Investigación Sanitaria Hospital 12 de Octubre (IMAS12), 28041 Madrid, Spain; 3 Centro de Investigación Biomédica en Red de Enfermedades Cardiovasculares (CIBERCV), 28041 Madrid, Spain; 4 Cardiology Department, Hospital Universitario Álvaro Cunqueiro, 36312 Vigo, Spain; 5 Cardiovascular Surgery Department, Hospital Universitario12 de Octubre, 28041 Madrid, Spain; 6 Radiology Department, Hospital Universitario 12 de Octubre, 28041 Madrid, Spain; 7 Facultad de Medicina, Universidad Complutense de Madrid (UCM), 28040 Madrid, Spain

**Keywords:** chronic thromboembolic pulmonary hypertension, balloon pulmonary angioplasty, pulmonary hypertension, pulmonary endarterectomy

## Abstract

(1) Background: Pulmonary endarterectomy (PEA) is the “gold standard” treatment for operable patients with chronic thromboembolic pulmonary hypertension (CTEPH). Persistent pulmonary hypertension (PH) after PEA confers a worse prognosis. Balloon pulmonary angioplasty (BPA) could represent a useful therapy in this setting, but evidence about its effectiveness and safety in patients with previous PEA is limited. (2) Methods: A total of 14 patients with persistent PH after PEA were treated with BPA in a single PH center. Hemodynamic and clinical effects of BPA and complications of the procedure were retrospectively collected. (3) Results: After BPA, the mean pulmonary arterial pressure fell from 50.7 ± 15.3 mmHg to 38.0 ± 7.9 mmHg (25.0% decrease; 95% confidence interval (CI) 14.0–35.5%; *p* = 0.01). Pulmonary vascular resistances were reduced from 8.5 ± 3.6 WU to 5.3 ± 2.2 WU (37.6% decrease; 95% CI 18.8–56.5%; *p* = 0.01). WHO functional class was also improved with BPA. Severe BPA-related complications were infrequent and no periprocedural deaths were observed. (4) Conclusions: BPA is an effective and safe therapy for patients with CTEPH and persistent PH after PEA.

## 1. Introduction

Chronic thromboembolic pulmonary hypertension (CTEPH) is caused by organized thrombi obstruction and distal pulmonary vasculopathy. It appears in up to 3.2% of pulmonary embolism survivors being the most important entity in group 4 pulmonary hypertension (PH) [1]. Pulmonary endarterectomy (PEA) is considered the “gold standard” treatment for CTEPH, being usually a curative treatment [2]. However, 5–35% of patients have residual pulmonary hypertension after PEA, which confers them with a worse prognosis [3].

Balloon pulmonary angioplasty (BPA) has emerged as the treatment of choice for non-operable CTEPH patients, with excellent hemodynamic and clinical results [4]. Patients with residual PH after PEA are an interesting subgroup where this therapy could be useful. Although relatively scarce, data in the literature support the hemodynamic and functional benefits of BPA in post-PEA patients [5,6,7,8,9,10,11]. However, information about the safety of BPA in this subset is even more limited and a higher rate of complications, such as vascular perforation, has recently been described [8].

Therefore, we aimed to analyze the effectiveness and safety of BPA in patients with residual PH after PEA treated at a PH reference center.

## 2. Materials and Methods

### 2.1. Study Patients

**Figure 1:**
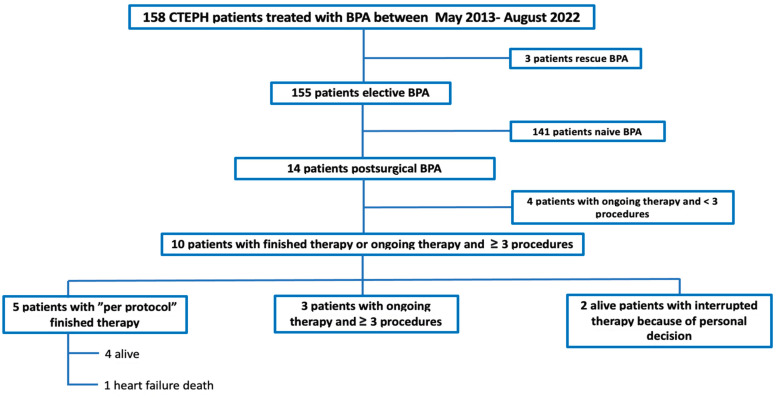
Flowchart of patients included in the study.

A descriptive, retrospective series study was conducted. Between May 2013 and August 2022, 263 patients underwent PEA and 158 CTEPH patients were managed with BPA at our center. From the total of 158 BPA patients, we excluded three patients where BPA was performed in a “rescue” indication because of cardiogenic shock, one of them being a postsurgical patient. We also excluded 141 “naive” patients in which no previous PEA was performed. The remaining 14 patients were included in the analysis. For the effectiveness analysis, only patients with finished therapy or with ongoing therapy and ≥3 BPA procedures were included; a total of 10 patients. For the safety analysis, all 14 postsurgical patients were included. The patients’ flowchart is displayed in Figure 1.

PEA was decided by a multidisciplinary team consisting of cardiac surgeons, PH cardiologists, pneumologists, BPA interventional cardiologists and radiologists. It was performed according to the San Diego technique in all patients [12]. Six months after surgery, a routine right heart catheterization (RHC) was conducted to evaluate the presence of residual PH. Although the cut-off value remains controversial, we defined residual PH as pulmonary vascular resistances (PVR) > 425 dyn·s·cm^−5^ (5.2 WU), as it is known that patients with PVR above this value after surgery show a worse prognosis [3]. If the patient had symptomatic residual PH despite double oral therapy with pulmonary vasodilators and had persistent thrombotic material amenable to be treated with angioplasty, BPA was considered. In these cases, a selective pulmonary angiography was performed as, in our experience, computed tomography diagnostic accuracy is lower in this setting [13]. The decision of being included in the BPA program was taken by the same multidisciplinary team that decided the initial surgery.

All patients were informed about their treatment options and possible risks associated with BPA. All of them provided written consent and agreed to the processing of their data. This study complied with the declaration of Helsinki for human research and was approved by the local ethics committee (number of research ethics committee approval 2014/0381).

### 2.2. Balloon Pulmonary Angioplasty Procedure

We performed BPA according to our previously described protocol [4]. All procedures were performed by three senior interventional cardiologists with experience in the treatment of patients with PH and substantial knowledge of the pulmonary vascular tree.

Prior to BPA, oral anticoagulation was stopped and the patient received low molecular weight heparin (LMWH) for 48 h. The last scheduled dose of LMWH before BPA was not administered. During the procedure, the patient was conscious and without sedation. BPA therapy included the treatment of all amenable lesions in all lobes, with the purpose of normalization of hemodynamic parameters and functional class. The first procedures were focused on the lower lobes since they are more vascularized. To avoid the development of reperfusion edema (RPE), we limited dilatations to two or three segmental branches of a single lobe per procedure while the mean pulmonary arterial pressure (mPAP) was >35 mmHg. Webs, bands, ring-like and pouch lesions were treated. We used 0.014” polymeric, low-tip-load wires such as Pilot 50^®^ (Abbott, Chicago, IL, USA) to cross the stenosis. Dilatation began with undersized balloons, followed by balloons of increasing sizes up to a maximum of 80% of the vessel size. Vessel size was estimated visually using selective pulmonary angiography. Intravascular ultrasound, optical coherence tomography, and pressure wires were not routinely used and were only employed in cases involving doubts about the lesion’s significance or to clarify the diagnosis of CTEPH [14]. Dilatation was considered effective when the distal flow improved, contrast uptake in the lung tissue increased and pulmonary venous return flow improved. We monitored patients for the development of RPE or pulmonary injury using chest X-rays obtained 8 h after each procedure. Patients continued hospitalization for 24–48 h to monitor complications.

### 2.3. Definitions

We considered that the BPA program was completed when all balloon angioplasty-amenable lesions of all lobes had been dilated. The BPA program was considered to be interrupted when any reason caused the therapy to be discontinued before achieving complete treatment of all possible lesions. We analyzed the data from an intention-to-treat perspective, considering that patients had finished BPA therapy when they completed the program according to the protocol or when they prematurely discontinued the BPA program for any reason.

Lung opacities after the procedure in the treated lobe/s were considered to indicate reperfusion edema (RPE), which has been recently renamed as pulmonary injury. To classify RPE, a modified version of Inami’s classification was used [15,16], which also took into consideration the clinical impact of radiological findings.

Based on angiographic findings, vascular injury during the procedure was classified as perforation which showed contrast extravasation, or vascular dissection.

Hemoptysis was classified as severe if it needed respiratory support or required any non-pharmacological intervention (balloon inflation or covered stent implantation) to stop it and mild if it did not meet the criteria for severe hemoptysis.

Periprocedural mortality was defined as the death of a patient that occurred 72 h following a BPA session or death during the index BPA hospital admission that was related to the BPA procedure.

Acute renal failure was defined according to the KDIGO criteria [17].

### 2.4. Clinical Assessment during Follow-Up and Complications

All patients underwent a standardized assessment prior to BPA. During therapy, hemodynamic variables were evaluated in each procedure, as an RHC was performed prior to BPA. After finishing the BPA program, patients were evaluated at 6 months, 12 months, and yearly afterward. WHO functional class (FC), 6-min-walking test (6MWT), serum levels of N-terminal prohormone of brain natriuretic peptide (NT-proBNP), and PH-specific medical therapy were assessed. An RHC was also performed at each scheduled visit. Mortality and heart failure hospitalization data were also collected during follow-up. In patients with completed therapy, the information of the RHC performed 6 months after finishing BPA therapy was used for the statistical analysis. In patients with ongoing therapy, the last hemodynamic parameters were evaluated.

We recorded procedural complications of all patients including hemoptysis, vascular injury, RPE, contrast allergy, acute renal failure and periprocedural mortality.

Data completeness was 100% for every variable except for 6MWT which was 90%.

### 2.5. Statistical Analysis

Statistical analysis was performed using the software STATA 16 (StataCorp, College Station, TX, USA). The normal distribution of quantitative variables was evaluated using the Shapiro–Wilk test. Results with a normal distribution were displayed as mean ± standard deviation. All other variables were expressed as median (interquartile range; IQR). Differences in quantitative variables obtained at baseline and post-BPA were analyzed with a Student’s t-test for paired data or the Wilcoxon test. Categorical or ordinal variables were compared using the McNemar or sign test for paired data. A value of *p* < 0.05 was considered statistically significant.

## 3. Results

Between May 2013 and August 2022, 263 patients underwent PEA in our center, of whom 34 had residual PH according to our definition. BPA was considered appropriate and suitable in 14 out of 34 patients and conformed to the study population. We performed a total of 50 procedures. The mean age was 49.2 ± 11.8 years, 70% being women.

The median time from PEA to the first BPA procedure was 2.1 years (IQR: 1.2–3.3). Ten of these patients had finished the therapy or had received ≥3 BPA procedures at the moment of the analysis and were included in the effectiveness evaluation. The mean number of procedures in these patients was 4.4 ± 2.0 procedures/patient. The mean contrast dose, fluoroscopy time and product dose area by procedure were, respectively, 320 ± 117 mL, 42 ± 16 min and m 173 ± 69 Gy*cm^2^. Baseline characteristics and procedural data are presented in Table 1. The evolution of the main hemodynamic variables before surgery, six months after surgery and before starting the BPA therapy is summarized in Table 2.

### 3.1. Effectiveness Analysis

**Figure 2:**
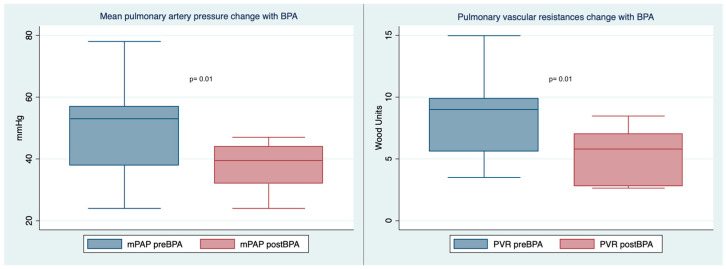
Box plot with mPAP (**left**) and PVR (**right**) changes after BPA. (BPA: balloon pulmonary angioplasty; mPAP: mean pulmonary artery pressure; PVR: pulmonary vascular resistance).

Table 3 shows hemodynamic and clinical variables before and after BPA treatment. BPA therapy was associated with a significant reduction in mPAP (mean reduction, 12.7 mmHg; 95% CI, 3.6–21.8; *p* = 0.01) and PVR (mean reduction, 3.2 WU; 95% CI, 0.8–5.6; *p* = 0.01) (Figure 2). No differences were observed in cardiac index (CI) and right atrial pressure (RAP).

**Figure 3:**
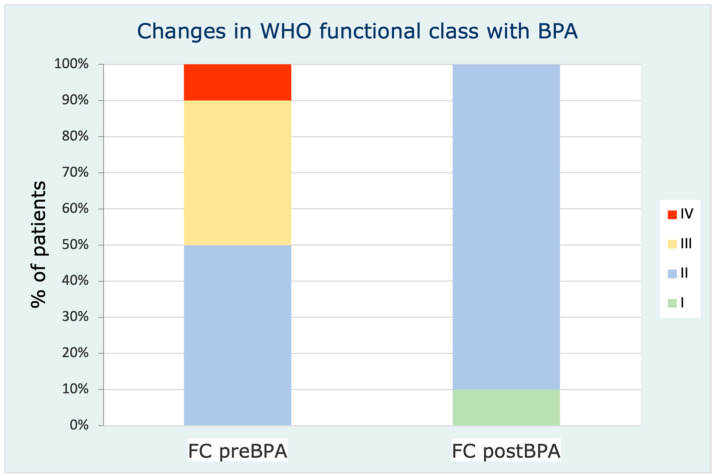
WHO functional class changes after BPA. (BPA: balloon pulmonary angioplasty; FC: functional class; WHO: world health organization).

The patients’ FC also improved significantly (Figure 3). Although a numerical trend was observed, no statistically significant differences were observed in the 6MWT, NT-proBNP, or the total number of PH-specific drugs. However, BPA allowed the stopping of intravenous prostanoids in all patients who were receiving them before BPA (five patients).

### 3.2. Safety Analysis

BPA-related complications are shown in Table 4. As previously explained, all 14 postsurgical patients were included in the safety analysis. The most frequent complication was hemoptysis (28.6% of patients and 14.0% of procedures) being all the episodes mild. RPE appeared in 21.4% of patients and 8.0% of procedures, none of them > grade 3. No vascular perforations were present. There was no periprocedural mortality.

With a median follow-up of 2.9 years (IQR: 1.8–3.4) only one patient died during hospitalization for acute heart failure. She was a 47-year-old woman with multiple comorbidities and persistent severe pulmonary hypertension (mPAP 44 mmHg, PVR 10 WU) two years after BPA and died because of cardiogenic shock in the context of a possible respiratory infection. No other cardiovascular events were present in the whole cohort of postsurgical patients.

## 4. Discussion

According to our findings, BPA in patients with symptomatic residual PH after PEA is proven to be effective and safe. BPA post-PEA was associated with a significant reduction in mPAP and PVR and an improvement in FC, all of that with a low incidence of severe complications.

PEA remains the “gold standard” treatment for patients with CTEPH. However, 5–35% of patients have persistent or recurrent PH after surgery. In observational studies, patients with mPAP > 38 mmHg or PVR > 425 dyn*s/cm^−5^ exhibit a worse prognosis with higher mortality during follow-up [3]. Although factors related to persistent PH after PEA are unknown, it may result from incomplete removal of thrombotic material from proximal pulmonary arteries, distal disease nonamenable for surgical extraction, pulmonary microcirculatory vascular remodeling, or recurrent thrombosis. Usually, a second PEA is not a possible option as the operative risk is too high or there is no additional proximal thrombotic material to be removed. Medical treatment with pulmonary vasodilators is a useful therapy for the treatment of microcirculation vasculopathy. However, it is sometimes insufficient to restore the hemodynamic abnormalities and control the patient’s symptoms. Additional “mechanical” therapies such as BPA, in the case of residual thrombotic material, have better efficacy with a higher reduction in PVR and improvement in functional class [18].

In this sense, our series showed a reduction of 25% in mPAP and 38% in PVR with no significant changes in RAP and CO. WHO FC was also better after BPA. Probably limited by sample size, a numerically but not statistically significant improvement in NT-proBNP (57% reduction) and 6MWT (26 m increase) was observed. Moreover, although not a significant reduction in PH-specific drugs was reached, all patients with intravenous prostanoids could cease the therapy after BPA. This fact reinforces the patient´s risk-profile reduction obtained with BPA.

Available experience about the effects of BPA in patients with persistent PH after PEA is still scarce in the literature, with a total number of patients of around 150, mostly coming from descriptive studies in Japan [5,6,8,11], where PEA is less frequently performed than in European/American countries and thus, experience with BPA is higher. The first data regarding the efficacy of BPA after PEA were published by Shimura et al. [5]. They studied nine patients with persistent PH after PEA, demonstrating that hemodynamics (PVR and mPAP decreased, no change in CO) and functional class improved after BPA. No differences were found in NT-proBNP and 6MWT. These findings were corroborated by similar results in other observational studies from Japan and Europe [6,7,8,9,10,11]. These studies even suggested that the efficacy of BPA is similar in post-PEA and naive BPA patients.

In our study, although we included patients with advanced but incomplete BPA therapy, we found similar results to the previously described in the European series [7,9,10] and slightly worse than the Japanese ones. Given that the published experience with BPA post-PEA is scarce to date, we consider that our data, coming particularly from a European setting, is important, adding useful information to settle the use of BPA in a challenging scenario such as persistent-PH after PEA, where its role is not clearly consolidated.

One interesting point is the optimal timing to perform BPA after surgery. Specifically, in our cohort, the median time between PEA and BPA was 2.1 years (IQR: 1.2–3.3), as most of the patients had longstanding persistent PH before post-PEA BPA was performed in our center. Although improvement of exercise capacity has been described until 2 years after PEA, a long time between PEA and BPA, with prolonged exposure to high pulmonary pressures, could promote vascular remodeling, which would advocate for early intervention. In fact, in patients who will course with residual PH, the benefit of surgery does not evolve after the first 3 months post-PEA [19,20]. For this reason, as included in our local protocol, a follow-up RHC 3–6 months after PEA is mandatory, to detect patients with persistent PH. Although medical therapy is the most used option in the case of persistent PH, we consider that according to the effectiveness of BPA, if a residual thrombus is present, early BPA after surgery may result in a more effective alternative to reduce PH and improve the patient´s risk situation.

Regarding procedural safety, our data showed a low incidence of severe BPA-related complications in post-PEA patients. The most common complication in postsurgical patients was hemoptysis, which occurred in 14% of procedures, followed by RPE in 8%. All episodes of hemoptysis and RPE were mild and did not require ICU support or interventional treatment. There was no perforation or need for embolization. Our results remark the good outcomes after BPA as no periprocedural deaths and only one death at follow-up was found in post-PEA patients of our cohort.

Published data about complications of BPA in post-PEA patients is as limited as the information regarding effectiveness. Some of the series have suggested an increased number of complications in postsurgical patients compared to naive ones (26% vs. 12%) [10]. However, the difference was mainly driven by mild to moderate complications. In our study, although a comparative approach was not the purpose and the anatomical/clinical characteristics of the patients might be different, we found a similar incidence of complications to the previously published in our initial cohort of naive BPA patients [4]. We find the frequent presence of vessel perforation described in most series particularly remarkable, with a reported incidence of 8–10%, probably related to vascular wall weakness after PEA [5,6,7,9,10]. In this context, especially concerning is the high incidence of severe hemoptysis requiring embolization (16% of patients) reported by Ito et al. [8]. Nevertheless, it is important to remark that the periprocedural mortality described in the literature is low, around 1% [5,6,7,8,9,10,11], and not different from the one in non-PEA patients. As previously mentioned, we had no significant perforation or requirement of distal embolization, nor periprocedural mortality. Thus, our data support the low incidence of severe complications remarking the safe profile of BPA in post-PEA patients. Although our results, particularly regarding vessel perforation, partially conflict with some of the previously published experiences, no definite explanation can be given for these differences between the series. The different definitions of complications in each observational study and technical differences in how the BPA procedure is performed may have an important role in these discrepancies.

**Figure 4:**
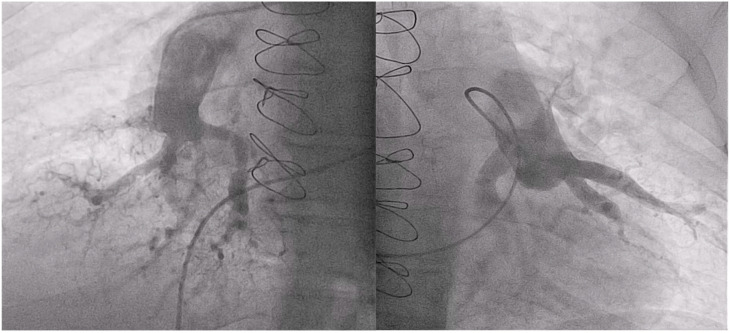
Pouch-like lesions with occlusion of segmental branches in both inferior lobe arteries of a postsurgical patient treated with BPA.

BPA for the treatment of patients with CTEPH is a demanding procedure with potentially life-threatening complications such as vascular perforation, hemoptysis or reperfusion edema. Moderate to severe adverse events are observed in approximately 10% of interventions and periprocedural mortality is described in around 2.5% [21]. The technical performance of BPA after PEA is usually more laborious and has particular characteristics such as postoperative changes in pulmonary vessels and anatomical distortion, making it more difficult to access the residual thrombotic material. These patients usually have very hard tortuous, calcified and multiple pouch-type lesions (Figure 4). Vessels are often weakened due to surgical techniques which lead to aneurysms. All these characteristics make crossing the lesions and catheter manipulation very challenging, increasing the potential risk of complications [22]. As an example of this, in our center’s experience, higher fluoroscopy time and radiation dose are needed than in regular BPA procedures. Thereby, in our cohort, the mean fluoroscopy time per procedure in PEA vs. non-PEA was 42 vs. 36 min and the median PDA was 173 vs. 86 Gy*cm2 [4].

All the available data highlight the efficacy and safety of BPA in a postsurgical setting. However, because of potential complications, a high level of technical experience is essential to avoid and treat the severe ones. In our opinion, to increase safety and efficacy, a BPA program in postsurgical patients must only be started when the learning curve for naive BPA is completely finished. In our institution, the postsurgical BPA program was started 5 years after the first BPA procedure, when more than 250 BPA procedures had already been performed.

## 5. Limitations

This was a single-center descriptive study with a small sample size. Limited statistical power may have precluded finding statistically significant differences. Enrolling patients with advanced but unfinished BPA therapy could have minimized the benefits of BPA in this subset of patients. However, the number of procedures was similar between patients with unfinished therapy and those who “per protocol” completed it (4.0 vs. 4.6 procedures).

## 6. Conclusions

Our data add new evidence that supports BPA as an effective therapy in CTEPH patients with residual PH after PEA, being associated with significant hemodynamic and functional improvement. Moreover, our work reinforces the safety of BPA in post-PEA patients, with a low rate of severe procedure-related complications and periprocedural mortality.

## Figures and Tables

**Table 1:** Baseline characteristics in postsurgical patients (*n* = 10).

Age (Years)	49.2 ± 11.8
Women (%)	70.0
Body mass index (Kg/m^2^)	25.9 ± 2.8
Thrombophilia (%)	30.0
Cancer history (%)	40.0
Hypothiroidism (%)	20.0
number of PH specific drugs	2.2 ± 0.6
Intravenous prostanoids (%)	50.0
Diuretics (%)	100.0
O_2_ (%)	30.0
N° of procedures/patient	4.4 ± 2.0
N° of treated lobes/procedure	1.3 ± 0.6
N° of treated segmentary arteries/procedure	2.6 ± 1.2
N° of treated subsegmentary arteries/procedure	4.4 ± 2.4
Balloon diameter (mm)	2.6 ± 0.9
Right atrium pressure (mmHg)	8.1 ± 5.3
Mean pulmonary artery pressure (mmHg)	50.7 ± 15.3
Cardiac index (L/min/m^2^)	2.8 ± 0.4
Pulmonary vascular resistances (W.U.)	8.5 ± 3.6
Pulmonay capilar wedge pressure (mmHg)	10.7 ± 7.3
Peripheric O_2_ saturation (%)	94.2 ± 3.3
Pulmonary artery O_2_ saturation (%)	66.7 ± 4.3

**Table 2:** Evolution of hemodynamic variables in post PEA patients (*n* = 10).

	Before PEA	After PEA	Before BPA	*p* before vs. after PEA	*p* after PEA vs. before BPA
Right atrium pressure (mmHg)	11.0 ± 3.9	10.0 ± 4.3	8.1 ± 5.3	0.41	0.06
Mean pulmonary pressure (mmHg)	54.0 ± 8.4	51.2 ± 8.3	50.7 ± 15.3	0.23	0.89
Cardiac index (l/min/m^2^)	2.7 ± 0.5	2.7 ± 0.8	2.8 ± 0.4	0.88	0.75
Pulmonary vascular resistances (W.U.)	9.7 ± 4.4	8.6 ± 2.9	8.5 ± 3.6	0.42	0.96
PEA: Pulmonary endarterectomy; BPA: Balloon pulmonary angioplasty.					

**Table 3:** Response variables pre and postBPA in postsurgical patients (*n* = 10).

	Before BPA	After BPA	*p*
Right atrium pressure (mmHg)	8.1 ± 5.3	8.3 ± 3.4	091
Mean pulmonary artery pressure (mmHg)	50.7 ± 15.3	38.0 ± 7.9	0.01
Cardiac index (L/min/m^2^)	2.8 ± 0.4	2.7 ± 0.7	0.75
Pulmonay vascular resistances (W.U.)	8.5 ± 3.6	5.3 ± 2.2	0.01
Six-minute walk test (m)	380 ± 118	407 ± 127	0.17
NT-proBNP (pg/mL)	680 (255–892)	292 (122–755)	0.21
number of PH specific drugs	2.2 ± 0.6	2.0 ± 0.8	0.51
Intravenous prostanoids (%)	50.0	0	0.02
number of PH specific drugs (%)			
0	0	0	
1	10.0	30.0	
2	60.0	40.0	
3	30.0	30.0	1
WHO functional class (%)			
1	0	10.0	
2	50.0	90.0	
3	40.0	0	
4	10.0	0	0.03

**Table 4:** Procedural complications.

	Per Patient (*n* = 14)	Per Procedure (*n* = 50)
Reperfusion edema (%)	21.4	8.0
grade 2	21.4	6.0
grade 3	7.1	2.0
grade 4	0	0
grade 5	0	0
Hemoptysis (%)	28.6	14.0
mild	28.6	14.0
severe	0	0
Vascular dissection (%)	21.4	6.0
Vascular perforation (%)	0	0
Contrast allergy (%)	7.1	2.0
Acute renal failure (%)	0	0
Periprocedural mortality (%)	0	

## Data Availability

Data supporting reported results can be consulted through mail to nicolasmmaneiro@gmail.com.
